# Recombination and amino acid point mutations in VP3 exhibit a synergistic effect on increased virulence of rMDPV

**DOI:** 10.1080/21505594.2024.2366874

**Published:** 2024-06-13

**Authors:** Jianye Wang, Wanmei Li, Xiaoyan Gong, Zhixian Wang, Yu Wang, Jueyi Ling, Zhiwei Jiang, Guoqiang Zhu, Yufeng Li

**Affiliations:** a Department of Preventive Veterinary Medicine, College of Veterinary Medicine, Yangzhou University, Yangzhou, Jiangsu, China; b Department of Preventive Veterinary Medicine, Jiangsu Co-Innovation Center for Important Animal Infectious Diseases and Zoonosis, Yangzhou, Jiangsu, China; c Shandong Academy of Agricultural Sciences, Institute of Poultry Science, Jinan, Shandong, China

**Keywords:** rMDPV, Muscovy duck parvovirus, goose parvovirus, recombination, rescue, virulence

## Abstract

Recombinant Muscovy duck parvovirus (rMDPV) is a product of genetic recombination between classical Muscovy duck parvovirus (MDPV) and goose parvovirus (GPV). The recombination event took place within a 1.1-kb DNA segment located in the middle of the VP3 gene, and a 187-bp sequence extending from the P9 promoter to the 5′ initiation region of the Rep1 ORF. This resulted in the alteration of five amino acids within VP3. Despite these genetic changes, the precise influence of recombination and amino acid mutations on the pathogenicity of rMDPV remains ambiguous. In this study, based on the rMDPV strain ZW and the classical MDPV strain YY, three chimeric viruses (rZW-mP9, rZW-mPR187, and rYY-rVP3) and the five amino acid mutations-introduced mutants (rZW-g5aa and rYY-5aa(ZW)) were generated using reverse genetic technology. When compared to the parental virus rZW, rZW-g5aa exhibited a prolonged mean death time (MDT) and a decreased median lethal dose (ELD_50_) in embryonated duck eggs. In contrast, rYY-5aa(ZW) did not display significant differences in MDT and ELD_50_ compared to rYY. In 2-day-old Muscovy ducklings, infection with rZW-g5aa and rYY-5aa(ZW) resulted in mortality rates of only 20% and 10%, respectively, while infections with the three chimeric viruses (rZW-mP9, rZW-mPR187, rYY-rVP3) and rZW still led to 100% mortality. Notably, rYY-rVP3, containing the VP3 region from strain ZW, exhibited 50% mortality in 6-day-old Muscovy ducklings and demonstrated significant horizontal transmission. Collectively, our findings indicate that recombination and consequent amino acid changes in VP3 have a synergistic impact on the heightened virulence of rMDPV in Muscovy ducklings.

## Introduction

Parvoviruses have a broad host range, infecting various organisms from vertebrates to arthropods. Muscovy duck parvovirus (MDPV) and goose parvovirus (GPV), belonging to the *Dependoparvovirus* genus within the *Parvovirinae* subfamily, are known to be pathogenic to waterfowl [1]. The genome of MDPV or GPV is a linear single-stranded DNA (ssDNA) of ~ 5.1 kb, and equal amounts of positive and negative strands are encapsidated in viral capsids [2]. The genome is flanked by identical inverted terminal repeats (ITRs), which comprise 414 ~ 456 nucleotides in various viral isolates [3–5]. ITR acts as the origin of genome replication and contains cis-acting elements, including the Rep-binding site (RBS), terminal resolution sites (TRS), and transcription factor-binding sites [2,6,7]. The P9 promoter, adjacent to the downstream of the 5′ ITR, transcribes one precursor (pre)-mRNA. Spliced P9 mRNA encodes the Rep1 protein, which has the largest molecular mass, and several small Rep proteins with low molecular masses [8,9]. Rep1 is involved in genome replication, virion assembly, and activation of the downstream P41 promoter. The P41 promoter lies inside the coding region of the carboxyl-terminus of Rep1 and transcribes cap-coding mRNA. Spliced P41 mRNA expresses three structural proteins, VP1, VP2, and VP3, through the use of different initiation codons [9]. VP1, VP2 and VP3 assemble a viral capsid at a ratio of 1:1:10 [9,10].

MDPV commonly infects Muscovy ducks (*Cairina moschata*) within three weeks of age, leading to Muscovy duck parvoviral disease, famously known as “three-week disease.” Clinical manifestations include diarrhea, lethargy, dyspnea, mental listlessness and mortality [11]. This disease is prevalent in Muscovy duck-raising regions such as China, France, and Japan, among others [12–15], with morbidity rates typically ranging from 27% to 62%, and mortality rates from 22% to 43%. In parallel with the classical Muscovy duck parvovirus, a distinct variant has emerged within Muscovy duck populations in China since the 1990s [16]. Notably, this variant exhibits a unique pathological feature of embolism formation in the intestinal tract, reminiscent of the pathology observed in goslings affected by GPV infection [17]. Unlike the classic “three-week disease,” this variant can pose a threat to Muscovy ducks up to 30 days of age, with mortality rates reaching approximately 60%. The culprit behind this disease is the recombinant MDPV (rMDPV) [18]. While much of the genetic makeup of rMDPV mirrors that of classical MDPV, a 1.1 kb region within the VP3 gene and a 187-bp segment covering the P9 promoter and upstream sequence of the Rep1 gene (referred to as the P9-rep sequence) have experienced recombination events with classical GPV [16,19,20]. This recombination resulted in the identification of five amino acid mutations in VP3, with four residing within the recombination region and one at the protein’s C-terminus. Furthermore, within the 187-bp P9-rep sequence, rMDPV integrates a homologous segment from SYG61v, an attenuated vaccine strain of GPV that is used for Derzsy’s disease prevention in China [5].

The specific contribution of the two recombination events and subsequent amino acid point mutations to the virulence of rMDPV remains unclear despite its genomic sequence. This study utilized reverse genetics technology developed in our laboratory to create a range of chimeric and mutant viruses. The pathogenicity of these engineered viruses was evaluated through propagation in embryonated Muscovy duck eggs and infection in ducklings. Our findings indicate that the combined effects of genome recombination and the resulting VP3 amino acid mutations play a synergistic role in enhancing the virulence of rMDPV in ducklings.

## Materials and methods

### Viral strains and infectious plasmid clones

The rMDPV strain ZW and the classical MDPV strain YY were previously isolated and preserved at −80°C in our laboratory. Their genome sequences were deposited in GenBank under accession numbers KY744743 and KX000918, respectively. Infectious plasmid clones pZW and pYY were generated earlier [16,21].

### Construction of chimeric plasmids pZW-mP9 and pZW-mPR187

**Figure 1.**
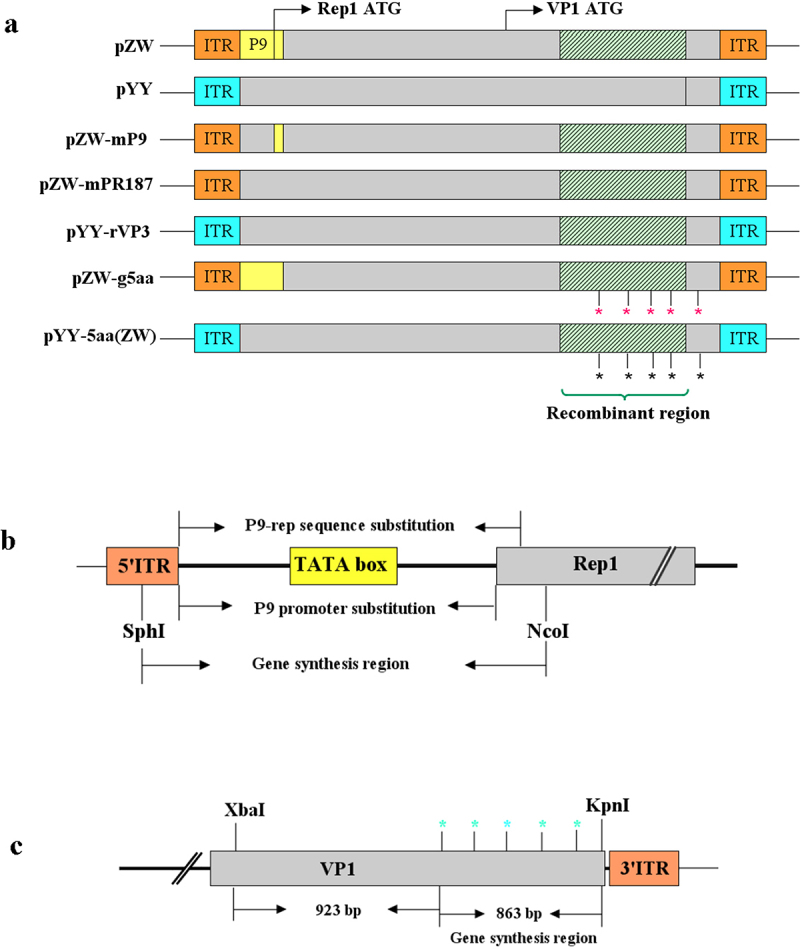
Illustration of the strategy used to construct chimeric or mutant plasmids. (a) Diagram showcasing the chimeric plasmids pZW-mP9, pZW-mPR187, pYY-rVP3, and the mutant plasmid pZW-g5aa, pYY-5aa(ZW). All these constructs were derived from the parental plasmids pZW and pYY, previously developed in our laboratory, containing the entire genome of the rMDPV strain ZW and the classical MDPV strain YY, respectively. The symbol * denotes the amino acid point mutation introduced into pZW or pYY. (b) Schematic diagram outlining the strategy for constructing chimeric plasmids pZW-mP9 and pZW-mPR187, wherein the P9 promoter or P9-rep sequence was replaced by the corresponding segment from the classical MDPV strain YY. (c) Schematic diagram illustrating the strategy for constructing the mutant plasmid pZW-g5aa or pYY-5aa(ZW) based on the parental plasmid pZW or pYY, involving the introduction of five amino acid mutations through a combination of gene synthesis and overlap PCR methods.

To investigate the impact of the recombinant P9-rep sequence on the pathogenicity of rMDPV, alterations were made to the original P9 promoter or P9-rep sequence in pZW by substituting them with the corresponding regions from the classical MDPV strain YY. To this end, chimeric plasmids pZW-mP9 and pZW-mPR187 were constructed using a gene synthesis strategy. Notably, a single *Sph*I site was situated within the middle loop of the ITR, while a sole *Nco*I site was identified at nucleotide position 721 (accession no.: 0KY744743). Two 500-bp DNA fragments containing *Sph*I and *Nco*I sites were synthesized (Genewiz, Soochow, China), incorporating the P9 promoter or P9-rep sequences from the classical MDPV strain YY (Figure 1(a,b)). Subsequently, these 500-bp DNA fragments were excised from the cloning vectors using *Sph*I and *Nco*I restriction enzymes for ligation processes. In previous studies conducted in our laboratory, the plasmids pBSKXBX-2.8 and pBSKXBX-2.3, encompassing the left 2.8-kb and the right 2.3-kb sub-genomic fragments of strain ZW, respectively, were established and employed to construct the infectious plasmid pZW [17]. pBSKXBX-2.8 was treated with *Sph*I and *Nco*I to release the 5.8-kb backbone. Subsequently, the two 500-bp DNA fragments were separately integrated into the 5.8-kb backbone, yielding the plasmids pBSKXBX-2.8(mP9) and pBSKXBX-2.8(mPR187). Following this, the pBSKXBX-2.3 plasmid underwent digestion with *Xba*I and *Bam*HI, enabling the isolation of the 2.3-kb subgenomic fragment, which was later purified and inserted into the *Xba*I/*Bam*HI cleaved pBSKXBX-2.8(mP9) and pBSKXBX-2.8(mPR187) plasmids. This step led to the development of the chimeric plasmids pZW-mP9 and pZW-mPR187, hosting the P9 promoter and 187-bp P9-Rep sequence from the classical MDPV strain YY, respectively.

### Construction of plasmid pZW-g5aa

rMDPV exhibited not only the integration of the corresponding 1.1-kb GPV fragment into the VP3 gene through recombination but also featured five distinct amino acid point mutations at positions 252, 287, 350, 387, and 514 within VP3. These mutations differ from those found in classical GPV and MDPV strains. Notably, among the five amino acid variations, residues 252, 287, 350, and 387 are situated within the 1.1-kb recombination area, while residue 514 is positioned downstream of this region. Specifically, residue 514 is notable as it is an asparagine in classical GPV and MDPV strains, whereas in rMDPV, it appears as a serine. Our objective was to generate plasmid pZW-g5aa to evaluate the pathogenicity of rMDPV in the context of recombination but without the presence of amino acid mutations.

To achieve this objective, the five amino acid sites in the pZW plasmid were modified to reflect the residues 252 (Asn→Ser), 287 (Pro→Thr), 350 (Ala→Thr), 387 (Arg→Thr), and 514 (Ser→Asn) observed in GPV. Utilizing a combination of overlap PCR and gene synthesis, the construction of pZW-g5aa was carried out (Figure 1(a,c)). Cloning was facilitated through the *Xba*I and *Kpn*I restriction sites situated at nucleotide positions 2818 and 4593. Initially, an 843-bp DNA fragment spanning the five targeted amino acid sites was synthesized (Genewiz, Soochow, China). Subsequently, a 1.7-kb fragment containing *Xba*I and *Kpn*I sites at both ends was amplified through overlap PCR, incorporating the 843-bp synthesized DNA fragment. This 1.7-kb fragment was integrated into a pMD19T vector (Takara, Dalian, China), leading to the formation of pMD19T–1.7. Upon digestion of pMD19T–1.7 with *Xba*I and *Kpn*I, the 1.7-kb DNA fragment was isolated, purified, and then linked with the *Xba*I-*Kpn*I-digested segment of pZW, ultimately resulting in the creation of the plasmid pZW-g5aa.

### Construction of plasmid pYY-5aa(ZW)

To further delve into the influence of the five amino acid mutations on the heightened virulence of rMDPV, we incorporated these mutations into the classical MDPV strain. The existing infectious plasmid pYY, encompassing the complete genome of the classical MDPV strain YY, was utilized for this purpose. Through gene synthesis and overlap PCR methods, we engineered pYY to create the plasmid pYY-5aa(ZW) (Figure 1(a,c)). The specific amino acid alterations introduced into the classical MDPV strain YY included residues 252 (Asn), 287 (Pro), 350 (Ala), 387 (Arg), and 514 (Ser), aligning with the corresponding amino acids observed in rMDPV.

### Construction of plasmid pYY-rVP3

To investigate the potential role of the recombinant VP3, incorporating both the 1.1-kb recombination region and the associated amino acid mutations, in the heightened virulence of rMDPV, we further constructed the chimeric plasmid pYY-rVP3 based on the parental plasmid pYY. To achieve the objective, we digested pZW with *Kpn*I and *Xba*I and excised the 1.7-kb DNA fragment encompassing the 1.1-kb recombination region along with the five characteristic amino acid sites. Simultaneously, the 6.3-kb backbone fragment was isolated from the pYY plasmid through *Kpn*I and *Xba*I digestion. The purified 1.7-kb and 6.3-kb DNA fragments were then ligated together, leading to the formation of the plasmid pYY-rVP3 (Figure 1(a)).

### Plasmids transfection and rescue

All the engineered plasmids were amplified in competent cells prepared with the *E*. coli strain SURE (Agilent, Santa Clara, USA). Subsequently, plasmids were purified using the TIANpure Mini Plasmid Kit (Tiangen, Beijing, China), and their DNA concentration and purity were assessed using a Nanodrop 2000 spectrophotometer (Thermo Fisher Scientific, USA). Plasmid transfection was conducted following established protocols [22], wherein each plasmid was mixed with Lipofectamine 2000 reagent (Invitrogen, Carlsbad, USA) at a ratio of 1:2.5 (μg:μl). The plasmid DNA-lipid complexes were infiltrated into the chorioallantoic membranes of 11-day-old embryonated Muscovy duck eggs (0.25 ml per egg, 2.0 μg plasmid). As a control, transfection with the vector plasmid pBluescript SK(+) was carried out. These eggs were candled three times daily and observed for 13 days. Number and time points (by hours) of embryo mortalities were documented. Upon observing mortality in the embryos due to viral infection 48 hours later post-transfection, allantoic fluid was collected. The rescued viruses were then diluted 1:50 in sterile saline and subsequently underwent two passages in 12-day-old embryonated Muscovy duck eggs. The genomes of the third egg passages were amplified using PrimeSTAR Max DNA polymerase (Takara, Dalian, China), and the resulting amplicons were purified for direct sequencing at Genewiz (Soochow, China).

### Determination of the medium egg lethal dose

In order to assess the viral titers of the rescued viruses, a duck embryo infection assay was conducted to determine the median embryo lethal dose (ELD_50_) following previously established procedures [22]. The second passages of the rescued viruses were utilized as the initial materials for ELD_50_ determination. Fresh allantoic fluid containing the virus was serially diluted in sterile saline to create a tenfold dilution series ranging from 10^−1^ to 10^−8^. For each dilution, five 12-day-old susceptible embryonated Muscovy duck eggs were inoculated with 0.2 ml of the virus via the allantoic cavity route. Subsequently, all embryos were examined under candling three times daily over a period of 7 days, and the number of embryo mortalities was documented. The ELD_50_ value was then calculated using the standard method delineated by Reed and Müench [23].

### Viral particles quantitation using real-time fluorescent PCR

The quantification of genomic copies in the allantoic fluid of the rescued viruses was conducted through quantitative real-time PCR (qPCR) using the ChamQ Universal SYBR qPCR master mix (Vazyme, Nanjing, China). To establish a standard plasmid template for qPCR, a 1.4-kb fragment of the Rep1 gene was amplified and cloned into the pMD19T vector (Takara, Dalian, China), generating the plasmid pMD-Rep1.4. This plasmid served as the reference for quantification in the qPCR assay. A set of qPCR primers targeting Rep1 were designed using Primer 5.0 software. The designed primers included an upstream primer 5’ATAACTAAAACCAAACGGGGAG 3’ and a downstream primer 5’ATAAAGCAGCAGCAGTGAAAAG 3.’ These primers were specific for amplifying a 136-bp DNA fragment from both the standard plasmid template and the viral DNAs. Viral DNA was extracted from the allantoic fluid using the TIANamp Genomic DNA kit (Tiangen, Beijing, China). The qPCR assays were performed in triplicate using an ABI 7500 real-time PCR system (Applied Biosystems, CA, USA) as per the manufacturer’s instructions.

### Infection of chimeric or mutant viruses in ducklings

The pathogenicity assessment of the chimeric and mutant viruses (rZW-mP9, rZW-mPR187, rZW-rVP3, rZW-g5aa, and rYY-5aa(ZW)), along with the parental viruses (rZW and rYY), was individually conducted in 2-day-old susceptible Muscovy ducklings. The infection experiments adhered to the ARRIVE guidelines (Animal Research: Reporting of In Vivo Experiments) available at https://arriveguidelines.org/. For the experiments, ducks were randomly assigned to eight groups, each consisting of 10 ducks. In the infection groups, the ducks were subcutaneously injected in the neck with the tested viruses at a dose of 0.5 ml containing 10^10^ viral particles. Ducks in the control group received an equivalent volume of sterile saline. Eight groups of ducks were housed in isolators with access to food and water *ad libitum*. Throughout a 14-day monitoring period, infected ducks were closely observed, and any deceased ducks underwent autopsy. Internal organs such as the liver, spleen, and intestine were harvested and homogenized. Following aseptic procedures, the tissue homogenates were utilized to inoculate 12-day-old embryonated Muscovy duck eggs for viral isolation. In cases where the embryos perished, allantoic fluid was pooled and further analyzed through PCR characterization and sequence analysis.

### Horizontal transmission test

Observations from clinical cases have indicated that rMDPV is pathogenic to Muscovy ducks up to the age of 20–30 days. In order to delve deeper into the role of the 1.1-kb VP3 recombination in the pathogenicity of rMDPV, the horizontal transmission potential of rYY-rVP3 was evaluated in 6-day-old Muscovy ducklings. Thirty 6-day-old Muscovy ducks were divided randomly into three groups consisting of 10 ducks each: infection group, horizontal contact group, and control group. The infection and horizontal contact groups were housed in separate cages that were positioned 30 cm apart within the same room, allowing free airflow between the cages. The control group was housed in a separate room. Ducks in the infection group were subcutaneously administered rYY-rVP3 (0.5 ml containing 10^10^ viral particles) in the neck. Those in the control group received an equivalent volume of sterile saline. All ducks in the three groups were provided open access to food and water throughout the experimental period, which spanned 20 days. Daily monitoring and recording of the ducks’ clinical signs were conducted, and any deceased ducks underwent post-mortem examination. Liver, spleen, and ileum tissues were collected for PCR characterization and viral isolation. At the conclusion of the experiment, surviving ducks were humanely euthanized through CO_2_ asphyxiation.

### Homology modeling of VP3

To investigate the impact of the five amino acid residues in the VP3 protein, three-dimensional structures of the VP3 proteins from strains ZW and rZW-g5aa were generated using the I-TASSER online server (https://zhanggroup.org/I-TASSER/), developed by Zhang et al. [24]. Homology modeling was performed utilizing the atomic structure of adeno-associated virus type 2 (AAV-2) (PDB: 1LP3) as a template. Subsequently, the predicted structures were analyzed using PyMOL (PyMOL Molecular Graphics System, Version 2.0, Schrödinger, LLC).

### Statistical analysis

Statistical analyses were performed using GraphPad Prism (GraphPad Software Inc., San Diego, CA, USA). Data are expressed as means ± standard deviation (SD). Survival analyses were conducted using the log-rank (Mantel-Cox) test. Group comparisons of MDT and ELD_50_ were performed using ordinary one-way ANOVA. Statistical significance was set at *p* < 0.05.

### Ethical statement

The procedures for the inoculation of ducks and embryonated duck eggs were ethically reviewed and approved by the Animal Care and Use Committee of Yangzhou University (approval number: SYXY-38). No additional permissions were required for conducting the experiments at these locations. As per the protocol, all living and sick ducks were humanely euthanized using CO_2_ throughout the experimental period.

## Results

### Generation of the chimeric and mutant infectious plasmids

rMDPV originates from dual recombination events-one within the middle region of the VP3 gene and the other involving the P9-rep sequence. Leveraging the parental infectious plasmids, pZW and pYY, three chimeric plasmids (pZW-mP9, pZW-mPR187, and pYY-rVP3) and two mutant plasmids (pZW-g5aa and pYY-5aa(ZW)) were developed (Figure 1(a)). Plasmids pZW-mP9 and pZW-mPR187 feature P9 promoter sequences identical to those of classical MDPV; however, they exhibit a disparity in the Rep1 ORF initiation region comprised of 95 nucleotides. Conversely, pYY-rVP3 possesses a VP3 sequence mirroring that of strain ZW, inclusive of the 1.1-kb recombination region, although its ITR and P9-rep sequences differ from strain ZW. Furthermore, pZW-g5aa bears five mutated amino acid positions sourced from GPV, while pYY-5aa(ZW) harbors five mutated amino acid positions characteristic of rMDPV.

### Virus rescue and passage in embryonated Muscovy duck eggs

**Figure 2.**
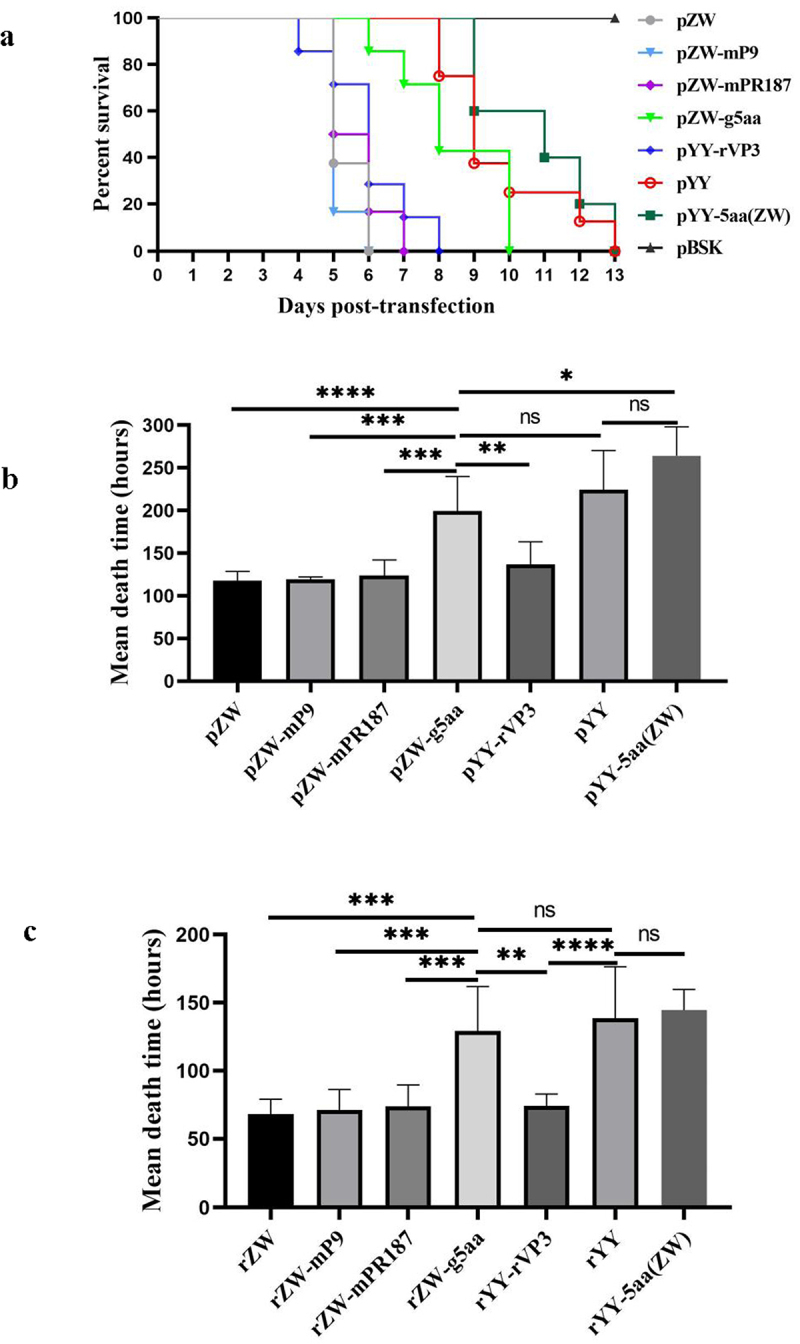
Survival curves and mean death times (MDT) in 11-day-old embryonated Muscovy duck eggs post-transfection or -infection of infectious plasmids or passaged viruses. (a) The survival curve of duck embryos transfected with the mutant plasmid pZW-g5aa significantly differed from those transfected with the parental plasmid pZW (p < 0.001), and the chimeric plasmids pZW-mP9 (p < 0.001), pZW-mPR187 (p < 0.01), and pYY-rVP3 (p < 0.05). However, similar survival curves were observed between duck embryos transfected with the mutant plasmid pYY-5aa(ZW) and the parental plasmid pYY (p > 0.05). No death occurred in duck embryos transfected with the vector plasmid pBSK until 13 days post-infection (dpi). (b) MDT of embryonated Muscovy duck eggs transfected with infectious plasmids. The MDT of pZW-g5aa-transfected duck embryos (199.4 hours) was significantly greater than that of pZW-mP9 (119.1 h) (p < 0.001), pZW-mPR187 (123.7 h) (p < 0.01), pZW (117.5 h) (p < 0.0001), and pYY-rVP3 (136.6 h) (p < 0.01)-transfected duck embryos. Conversely, the MDT of pYY-5aa(ZW)-transfected duck embryos (264 hours) was similar to that of the parental plasmid pYY (224.2 h) (p > 0.05). (c) The first-generation rescued viruses, diluted at 1:50, were individually passaged in 12-day-old Muscovy duck eggs. The MDT of rZW-g5aa (129.2 hours) was significantly greater than that of rZW-mP9 (71.2 h) (p < 0.01), rZW-mPR187 (73.8 h) (p < 0.05), rZW (68 h) (p < 0.01), and rYY-rVP3 (74.2 h) (p < 0.05). Conversely, the MDT of rYY-5aa(ZW)-inoculated duck embryos (144.5 hours) was similar to that of the parental virus rYY (138.5 h) (p > 0.05). Legend: ns, not significant; *p < 0.05; **p < 0.01; ***p < 0.001; and ****p < 0.0001.

After transfection with three infectious chimeric plasmids (pZW-mP9, pZW-mPR187, and pYY-rVP3), all duck embryos succumbed within 8 days (Figure 2(a)). The mean death times (MDT) for the duck embryos transfected with these plasmids were recorded as 119.1 hours, 123.7 hours, and 136.6 hours, respectively, closely resembling that of the parental plasmid pZW-transfected embryos (117.5 hours) (*p* > 0.05) (Figure 2(b)). In contrast, duck embryos transfected with pZW-g5aa experienced mortality by day 6 post-infection, exhibiting 100% mortality by day 11 (Figure 2(a)). The MDT for pZW-g5aa-transfected duck embryos was notably extended to 199.4 hours, significantly surpassing the MDTs of the three chimeric plasmids and pZW-transfected duck embryos (*p* < 0.01) (Figure 2(b)). Furthermore, the MDT for pYY-5aa(ZW)-transfected duck embryos was determined to be 264 hours, slightly longer than that of the parental plasmid pYY-transfected embryos (224.2 hours); however, the disparity was not statistically significant (*p* > 0.05).

Subsequently, the chimeric and mutant viruses were denoted as rZW-mP9, rZW-mPR187, rYY-rVP3, rZW-g5aa, and rYY-5aa(ZW), with the parental viruses named rZW and rYY. The propagation capabilities of the rescued viruses were gauged through passaging in 12-day-old embryonated Muscovy duck eggs, and the MDT of the second passage was evaluated and compared. The MDT for rZW-g5aa-inoculated duck embryos was determined to be 129.2 hours, significantly longer than that of rZW-mP9 (71.2 hours) (*p* < 0.001), rZW-mPR187 (73.8 hours) (*p* < 0.01), rYY-rVP3 (74.2 hours) (*p* < 0.01), and rZW (68 hours) (*p* < 0.001) (Figure 2(c)). The MDT of rYY-5aa(ZW) was calculated at 144.5 hours, closely aligning with those of rYY (138.5 hours) (*p* > 0.05) and rZW-g5aa (129.2 hours) (*p* > 0.05), but significantly longer than those of rZW-mP9 (*p* < 0.001), rZW-mPR187 (*p* < 0.001), rYY-rVP3 (*p* < 0.001), and rZW (68 hours) (*p* < 0.001).

Lastly, PCR amplification and sequencing confirmed the correct genomic sequences of the third passage. The genomic sequences of all rescued chimeric and mutant viruses were accurate. Notably, the genomic sequences of rZW and rYY mirrored those of the parental strains ZW and YY, respectively. Therefore, the findings underscored that the pathogenicity of the mutant virus rZW-g5aa in Muscovy duck embryos was markedly reduced compared to the parental virus rZW and the other chimeric viruses, showcasing the significance of the five mutated amino acids in the adaptation of rMDPV in embryonated Muscovy duck eggs.

### Comparison among the titers of the rescued viruses

**Figure 3.**
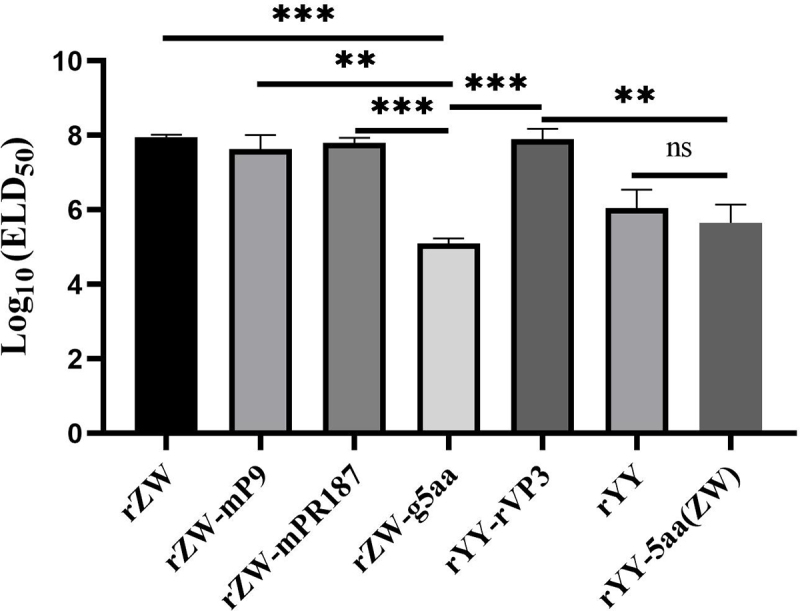
ELD_50_ of the second egg passages of the rescued viruses is compared. The mutant virus rZW-g5aa demonstrates an ELD_50_ of 10^5.10^/ml, significantly lower than that of the chimeric viruses rZW-mP9 (10^7.64^/ml) (p < 0.01), rZW-mPR187 (10^7.80^/ml) (p < 0.01), rYY-rVP3 (10^7.90^/ml) (p < 0.01), and the parental virus rZW (10^7.95^/ml) (p < 0.001). On the other hand, the mutant virus rYY-5aa(ZW), which carries the five characteristic residues of rMDPV, demonstrates a similar ELD_50_ (10^5.65^/ml) compared to the parental virus rYY (10^6.05^/ml) (p > 0.05).

In the second passage of the rescued viruses, the determination of their median embryo lethal dose (ELD_50_) was conducted, with average ELD_50_ values from two independent experiments utilized for comparison. The ELD_50_ values for the chimeric viruses rZW-mP9, rZW-mPR187, and rYY-rVP3 were gauged at titers of 10^7.64^/ml, 10^7.80^/ml and 10^7.90^/ml, respectively, closely resembling that of the parental virus rZW (10^7.95^/ml) (*p* > 0.05) (Figure 3). Conversely, the ELD_50_ for the mutant virus rZW-g5aa registered at 10^5.10^/ml, significantly lower than that of rZW (*p* < 0.001) and the three chimeric viruses. Meanwhile, rYY-5aa(ZW) exhibited an ELD_50_ of 10^5.65^/ml, akin to that of the parental virus rYY (10^6.05^/ml) (*p* > 0.05) and rZW-g5aa (*p* > 0.05), but notably lower than that of rZW-mP9, rZW-mPR187, rYY-rVP3, and rZW (*p* < 0.01). These findings underscored the substantial decrease in the propagation of rZW-g5aa in duck embryos due to the five amino acid mutations, whereas the acquisition of the same mutations did not enhance the propagation of rYY-5aa(ZW) compared to the parental strain rYY.

### The recombination occurred in VP3 gene plays a pivotal role in increasing the virulence of rMDPV in Muscovy ducklings

The pathogenicity of the chimeric and mutant viruses was further investigated in 2-day-old Muscovy ducklings through post-mortem examinations conducted on seven groups of deceased ducks. Organ tissues such as the liver, kidney, and ileum were collected from the deceased ducks for PCR amplification. Sequencing analysis of the amplicons confirmed that the viral sequences matched those of the respective challenged viruses.

**Figure 4.**
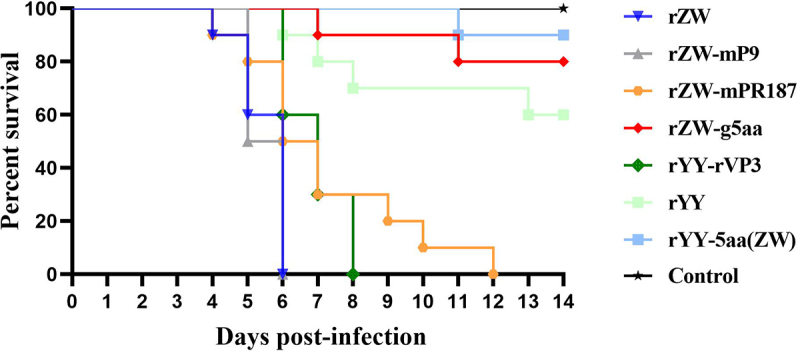
Survival curves of 2-day-old Muscovy ducklings infected with the rescued viruses, encompassing three chimeric viruses, two mutant viruses, and two parental viruses. The group infected with the mutant virus rZW-g5aa displayed a significantly distinct survival curve compared to the groups infected with chimeric viruses (rZW-mP9, rZW-mPR187, rYY-rVP3) and the parental virus rZW (p < 0.0001). Conversely, similar survival curves were noted between the group infected with the mutant virus rYY-5aa(ZW) and the parental virus rYY (p > 0.05). Despite witnessing 100% mortality in the groups infected with the three chimeric viruses and rZW, the survival curve of the rZW-mPR187 infection group displayed slight variability compared to the rZW-mP9 and rZW infection groups (p < 0.05), yet similarity to the rYY-rVP3 infection group (p > 0.05).

In the infection groups, rZW and rYY led to 100% and 40% mortality, respectively, within the 14-day observation period. All ducks in the rZW group succumbed within 6 days, while only four deaths were recorded in the rYY group at 6, 7, 8, and 13 dpi (Figure 4). These results indicated that rMDPV exhibited higher virulence in Muscovy ducklings compared to classical MDPV. The chimeric virus rYY-rVP3, developed from the backbone of classical MDPV strain YY, resulted in 100% mortality with all infected ducks perishing within 8 days. The survival curve of the rYY-rVP3 infected group significantly differed from that of the rYY infection group (*p* < 0.01), suggesting an enhanced virulence attributed to the VP3 recombination event.

Further evaluation of the five characteristic amino acids revealed that the mortality of ducks infected with rZW-g5aa was only 20%, with two deaths occurring at 7 and 11 dpi. The survival curve of the rZW-g5aa infection group significantly differed from that of the rZW, rZW-mP9, rZW-mPR187, and rYY-rVP3 infection groups (*p* < 0.0001). In contrast, the mortality of ducks infected with rYY-5aa(ZW) was only 10%, with a single death recorded at 11 dpi. The survival curve of the rYY-5aa(ZW) infection group was similar to that of the rYY and rZW-g5aa infection groups (*p* > 0.05), but significantly different from those of rZW, rZW-mP9, rZW-mPR187, and rYY-rVP3 (*p* < 0.0001). These findings highlight that integrating the five characteristic residues into classical MDPV does not enhance its pathogenicity in Muscovy ducklings. Instead, the VP3 recombination event in conjunction with the five characteristic amino acid mutations synergistically contributed to the increased virulence of rMDPV.

### The recombination of the P9-rep sequence plays a minor role in the increased virulence of rMDPV in ducklings

Similar to the chimeric virus rYY-rVP3, the other two chimeric viruses, rZW-mP9 and rZW-mPR187, also led to 100% mortality in infected ducks (Figure 4). In the case of rZW-mP9 and rZW infection groups, where all ducks succumbed within 6 days, a slight variation was observed in the rZW-mPR187-infected ducks as only half perished within 6 days, with the final death occurring at 12 dpi. The survival curves showed similarity between the rZW-mP9 and rZW infection groups (*p* > 0.05), while a noticeable difference was noted between the rZW and rZW-mPR187 infection groups (*p* < 0.05) as well as between the rZW-mP9 and rZW-mPR187 infection groups (*p* < 0.05). Despite the 100% mortality in the rYY-rVP3 infection group, the survival curve significantly differed from that of the rZW infection group (*p* < 0.01) and the rZW-mP9 infection group (*p* < 0.001), but was similar to that of the rZW-mPR187 infection group (*p* > 0.05).

It was observed that the sequences of P9-rep were identical in rYY-rVP3 and rZW-mPR187, with variations detected in their ITR sequences. Given the 98% identity in the ITR sequence between the rMDPV strain ZW and the classical MDPV YY strain, it was hypothesized that minor discrepancies in the ITR sequences played a minimal role in the increased virulence of rMDPV in ducklings. In summary, the study results suggest that recombination of the entire P9-rep sequence may play a role in enhancing the virulence of rMDPV to some degree. However, the substitution of the sole P9 promoter did not result in discernible pathogenic alterations in Muscovy ducklings.

### rYY-rVP3 demonstrates a significantly enhanced horizontal transmission capability

**Figure 5.**
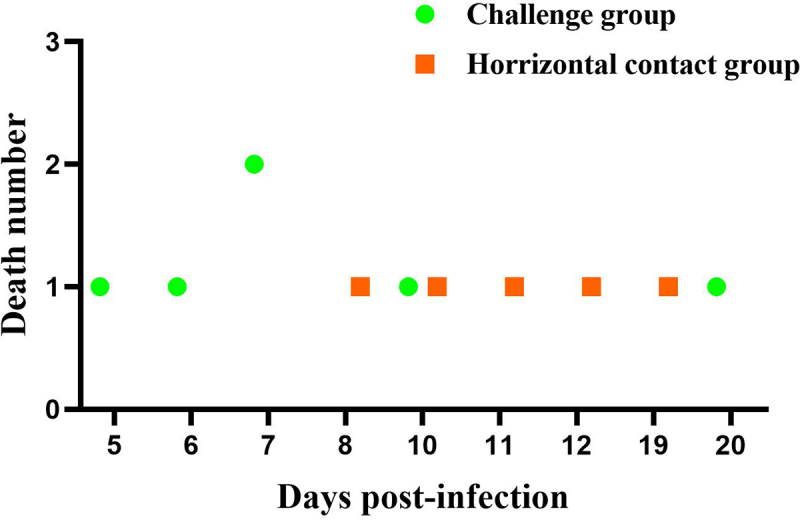
The pathogenicity and horizontal transmission capability of rYY-rVP3 were assessed using a 6-day-old Muscovy duck model. During the 20-day observation period, the challenge group displayed a 50% mortality rate, with the first death occurring at 5 dpi. In the horizontal contact group, the initial death was observed at 8 days, and cumulative mortality reached 50% within the observation period.

**Figure 6.**
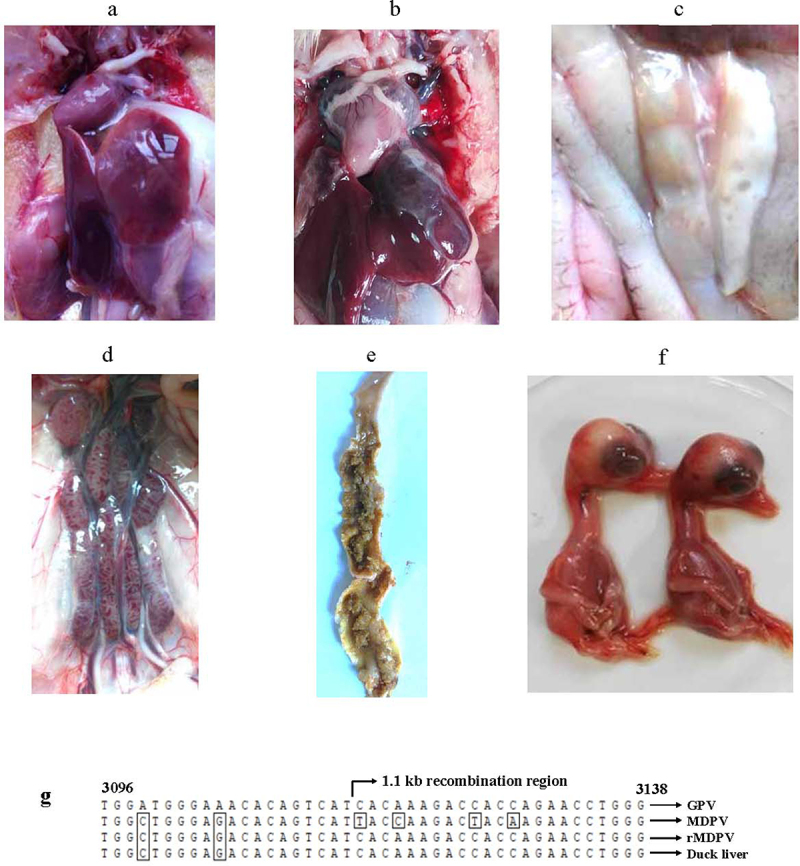
Postmortem examination of deceased Muscovy ducks in the horizontal contact group and viral isolation from tissue samples revealed several pathological signs. These included pericardial effusion (a), fibrous exudates on the liver surface (b), necrotic spots in the pancreas (c), congested kidney (d), and embolism formation in the intestine (e). (f) Liver tissue homogenates from the deceased ducks were used to inoculate 12-day-old embryonated Muscovy duck eggs, resulting in embryo death and the manifestation of hemorrhagic lesions in embryo bodies. (g) Viral DNA extracted from the pooled allantoic fluid was subjected to PCR characterization. Sequence analysis demonstrated 100% homology with the rMDPV strain ZW. Nucleotide numbering was based on the genome sequence of the classical GPV strain LH (accession number KM272560).

The impact of the 1.1-kb recombination region on the enhanced virulence of rMDPV was further examined through a horizontal transmission experiment involving 6-day-old Muscovy ducks. In the group infected with rYY-rVP3, six ducks died during the monitoring period, four of them between days 5 and 7, while the remaining two expired at 10 and 20 dpi (Figure 5). In the horizontal contact group, mortality occurred at 8, 10, 11, 12, and 19 dpi, resulting in a 50% mortality rate. Postmortem examination of the deceased ducks in the horizontal contact group revealed gross pathological abnormalities, including pericardial effusion, liver and kidney congestion, white fibrous exudates covering the liver surface, pancreatic necrosis dots, and discontinuous embolism in the intestine (Figure 6(a-e)). These pathological changes aligned with those observed in field cases associated with rMDPV infection.

Tissue homogenates from the horizontal contact groups were used for viral isolation in 12-day-old embryonated Muscovy duck eggs. The embryos succumbed between 96 and 130 hours, displaying hemorrhagic lesions (Figure 6(f)). The allantoic fluid from the deceased embryos was pooled for DNA extraction, PCR amplification, and sequencing. The generated amplicons exhibited 100% nucleotide identity with the challenged virus rYY-rVP3 (Figure 6(g)), confirming that duck mortality in the horizontal contact group was a result of rYY-rVP3 infection. Taken together, these findings demonstrate that the chimeric virus rYY-rVP3 demonstrates substantial horizontal transmission capacity in Muscovy ducklings older than six days, highlighting the crucial role of the recombinant VP3 event in the heightened virulence of rMDPV.

### Structural modeling of the five amino acid mutations

**Figure 7.**
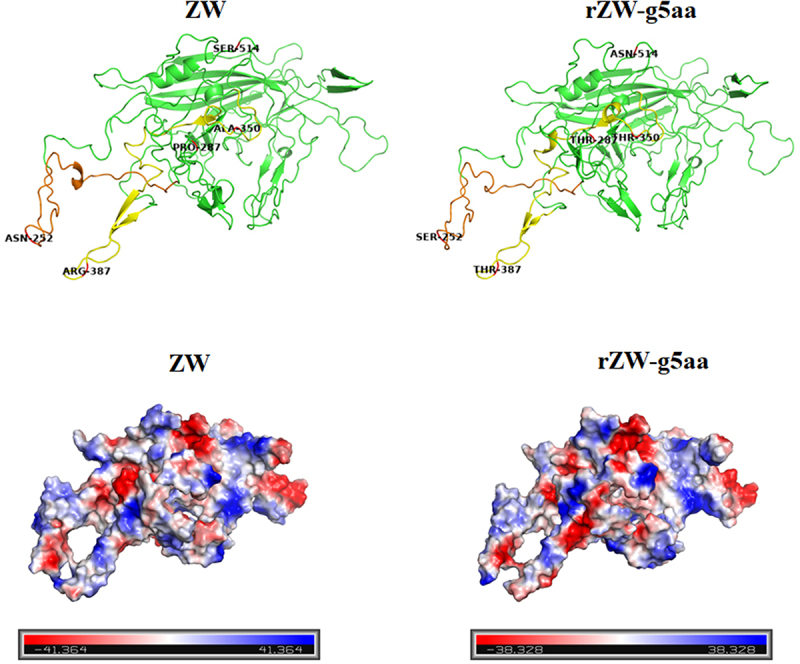
Three-dimensional structures and vacuum electrostatics of VP3 are presented. (a) Stereo diagrams illustrate the tertiary structures of VP3 proteins from strain ZW and the mutant virus rZW-g5aa. The mutated residues 252, 350, and 387, likely exposed on the exterior surface of the capsid, are located in two discontinuous peptide chains, which were highlighted in orange and yellow, respectively. (b) Electrostatic surfaces of VP3 proteins from strain ZW and rZW-g5aa demonstrate negative and positive charges represented in blue and red, respectively.

Homology modeling suggests that the five amino acids characteristic of rMDPV are predicted to reside in the fully exposed loops or random coils of VP3 (Figure 7(a)). Notably, residues 252, 350, and 387 are likely to be positioned on the exterior surface of the capsid, as indicated by previous analyses [25]. Additionally, the stereo diagram of the tertiary structure reveals a close spatial proximity between residues 252 and 387. The substitutions of these five amino acids in VP3 of rZW-g5aa not only induce minor alterations in the tertiary structure of VP3 but also modify the partial electrostatic distribution, leading to a decrease in the net positive charge of VP3 (Figure 7(b)). Consequently, these mutated residues play a pivotal role in promoting the assembly of viral capsids with heightened fitness among structural proteins, providing the structural foundation for the increased virulence of rMDPV. Conversely, in the context of rZW-g5aa harboring five amino acid mutations, significant virulence attenuation is anticipated due to the altered capsid structure.

## Discussion

Recombination can expedite viral evolution and may occur either between field strains or between field strains and vaccine strains, as highlighted in studies [25,26]. This process aids the virus in evading the protective effects of the original vaccine. The emergence of rMDPV stems from dual distinct recombination events: one within the central 1.1-kb DNA region of the VP3 gene and the other involving the 187-bp P9-rep sequence. Concurrent with the 1.1-kb DNA recombination event, five characteristic amino acid point mutations emerge in the VP3 protein, setting it apart from those found in classical GPV and MDPV [16]. This investigation aims to elucidate the influence of these two recombination events and the accompanying amino acid point mutations on the pathogenicity of rMDPV.

From the parental viruses rZW and rYY, two mutant viruses, rZW-g5aa and rYY-5aa(ZW), were generated to assess the role of the five characteristic residues in the increased virulence of rMDPV. Virulent strains of GPV or MDPV replicate inadequately in primary goose or duck embryo fibroblasts [27], thereby restricting their growth kinetics and titer comparison in cell culture. Both MDT and ELD_50_ were employed in this study to evaluate the propagation capability and titer variances among the rescued viruses. The differences in MDT and ELD_50_ were insignificant between rYY-5aa(ZW) and rYY but significant between rZW-g5aa and rZW. Although the rYY-5aa(ZW) and rYY infection groups displayed similar survival curves in the duck infection test, the mortality rate of the rYY-5aa(ZW) infection group was only 10%, lower than that of the rYY infection group. These findings indicate that solely introducing the five characteristic residues of rMDPV into classical MDPV does not suffice to enhance its propagation capability in duck embryos and its virulence towards Muscovy ducklings.

The creation of the mutant virus rZW-g5aa enabled an assessment of whether the 1.1-kb DNA recombination alone was the exclusive factor contributing to the heightened virulence of rMDPV. Upon culturing the mutant viruses in embryonated Muscovy duck eggs, it was observed that rZW-g5aa exhibited a prolonged mean death time (MDT) and reduced ELD_50_ compared to the parental virus rZW. Infection with rZW-g5aa led to only 20% mortality in ducklings, while 100% mortality was observed in rZW-infected ducks. Accordingly, the survival curve of the rZW-g5aa infection group significantly differed from that of the parental virus rZW but was similar to that of rYY. These results indicate that recombination alone is insufficient for rMDPV to acquire potent pathogenicity towards Muscovy ducklings; the presence of five characteristic amino acid point mutations is crucial for augmenting the virulence of rMDPV.

rMDPV underwent secondary recombination in the P9-rep region, detectable in viral isolates such as SAAS-SHNH, ZW, FJM3, JH06, JH10, 20-0910 G, and XMX [16,19,20]. Small-scale recombination led to the replacement of the original sequence with that of the GPV vaccine strain SYG61v. The P9-rep sequence comprises 187 nucleotides, encompassing the 92-bp P9 promoter and a 95-bp fragment upstream of the Rep gene. To assess the potential role of P9-rep sequence recombination in rMDPV pathogenicity, three chimeric viruses were generated: rZW-mP9, rZW-mPR187, and rYY-rVP3. rZW-mP9 and rZW-mPR187 carry the P9 promoter and P9-rep sequences, respectively, from classical MDPV. The MDT and ELD_50_ of rZW-mP9 and rZW-mPR187 closely resembled those of the parental strain rZW, indicating that P9-rep sequence recombination insignificantly affects the propagation capability of rMDPV in embryonated Muscovy duck eggs. Despite inducing 100% mortality in 2-day-old Muscovy ducks, rZW-mPR187 and rZW-mP9 exhibited similar virulence to the parental virus rZW. However, the survival curves of infected ducks significantly differed between rZW-mPR187 and rZW, and between rZW-mPR187 and rZW-mP9. Both rZW and rZW-mP9 infection groups experienced 100% mortality by 6 dpi, while the rZW-mPR187 infection group showed only 50% mortality by 6 dpi, with the last death occurring at 12 dpi. These outcomes indicate that solely replacing the P9 promoter does not notably affect rMDPV pathogenicity, whereas complete substitution of the P9-rep sequence results in a slight reduction in rMDPV virulence towards Muscovy ducklings. Consequently, these findings suggest that the entire P9-rep sequence, potentially encompassing the 95-bp DNA region at the N-terminus of Rep1, likely contributes to the heightened virulence of rMDPV. Studies on adeno-associated virus (AAV-2) have shown that the P5 promoter and the 5′-terminal sequence (nucleotide position 190–540) of the Rep gene can enhance genome replication and packaging [28]. Altogether, the current results indicate that P9-rep sequence recombination may play a minor role in driving the increased virulence of rMDPV, warranting further investigation into the underlying molecular mechanism.

rYY-rVP3 incorporates the complete VP3 gene from the rMDPV strain ZW into the backbone of the classical MDPV strain YY. Compared to rYY, rYY-rVP3 exhibited a lower MDT and higher ELD_50_, underscoring the significant impact of the recombinant VP3 gene on the increased adaptation of rMDPV in embryonated Muscovy duck eggs. Infection with rYY-rVP3 in 2-day-old ducks resulted in 100% mortality, whereas only 40% mortality was observed in rYY-infected ducks, indicating that the recombinant VP3 gene is the primary determinant of the augmented virulence of rMDPV in Muscovy ducklings. Despite sharing an identical P9-rep sequence with rZW-mPR187, rYY-rVP3 possesses 13 nucleotide differences in the ITR relative to the latter [16]. rYY-rVP3 and rZW-mPR187 showed similar ELD_50_ and MDT, and presented parallel survival curves during duckling infection, suggesting that minor sequence variations in the ITR do not account for the increased virulence of rMDPV. Moreover, despite the 100% mortality induced by rYY-rVP3 infection, discernible differences in survival curves were still observed between the rYY-rVP3 and rZW-mP9 infection groups, reinforcing the previous conclusion that P9-rep sequence recombination, rather than the sole P9 promoter replacement, contributes to the virulence enhancement of rMDPV.

Classical MDPV typically infects Muscovy ducks within three weeks of age, whereas rMDPV can pose a threat to Muscovy ducks up to 30 days old [18]. The contribution of recombinant VP3 to rMDPV pathogenicity was examined in a 6-day-old Muscovy duck model using a horizontal transmission test. rYY-rVP3 infection in the horizontal contact group led to 50% mortality, with the latest deceased duck being 25 days old. Postmortem examinations, viral isolation, and genome sequencing confirmed duck deaths in the horizontal contact group due to rYY-rVP3 infection. The robust horizontal transmission capability of rYY-rVP3 underscores the critical role of the 1.1-kb recombination region of VP3 in enhancing the virulence of rMDPV.

Furthermore, the role of the five characteristic amino acids in the capsid structure of rMDPV was assessed. Through homology modeling of rMDPV VP3 with AAV, it was found that the five amino acids are located at the loops, with residues 252 and 387 from neighboring loops proximate in the three-dimensional structure. Compared to the ZW parent strain model, the rZW-g5aa capsid exhibited perturbations in its three-dimensional structure and vacuum electrostatics. Loops typically represent the surface features of parvoviruses governing their interactions with cellular receptors [29,30]. According to a prior report by Bossis et al. [31], residues 252, 350, 387, and 514 are presumed to be exposed on the capsid′s exterior surface, indicating that mutations in these residues likely affect binding to the host cell receptor, potentially explaining the reduced pathogenicity of rZW-g5aa towards Muscovy ducks.

In summary, this study highlights that the 1.1-kb DNA recombination of the VP3 gene, rather than the P9-rep sequence recombination, plays a crucial role in the increased virulence of rMDPV. Specifically, the five characteristic amino acid point mutations in VP3, coupled with the 1.1-kb DNA recombination event, are essential for the acquisition of virulence in rMDPV. Among these five residues, four are situated within the 1.1-kb VP3 recombination region, while only one is in the near-carboxyl terminus of VP3. Thus, further elucidation of the amino acids pivotal to the heightened virulence of rMDPV is warranted.

## Data Availability

The data that support the findings of this study are openly available in figshare at http://doi.org/10.6084/m9.figshare.25416529, reference number 25,416,529.
